# Perspectives of Aboriginal women on participation in mammographic screening: a step towards improving services

**DOI:** 10.1186/s12889-017-4701-1

**Published:** 2017-09-11

**Authors:** Leanne Pilkington, Margaret M. Haigh, Angela Durey, Judith M. Katzenellenbogen, Sandra C. Thompson

**Affiliations:** 1 Aboriginal Health Strategy, WA Country Health Service, Department of Health, Level 7, 2 Mill Street, Perth, WA 6000 Australia; 2 BreastScreen WA, , 9th Floor, Eastpoint Plaza, 233 Adelaide Terrace, Perth, WA 6000 Australia; 3 Western Australian Centre for Rural Health, The University of Western Australia, 35 Stirling Highway, Perth, WA 6009 Australia; 4 School of Dentistry, The University of Western Australia M512, 35 Stirling Highway, Perth, WA 6009 Australia; 5 Centre for Aboriginal Studies, Curtin University, Perth, WA 6102 Australia; 6 School of Population and Global Health, The University of Western Australia M512, 35 Stirling Highway, Perth, WA 6009 Australia; 7 Western Australian Centre for Rural Health, The University of Western Australia, PO Box 109, Geraldton, WA 6530 Australia

**Keywords:** Aboriginal, Indigenous, Breast cancer, Breast screening, Mammography

## Abstract

**Background:**

Early detection of breast cancer using screening mammography provides an opportunity for treatment which can lead to significantly improved outcomes. Despite considerable efforts having been made, the rate at which Aboriginal and Torres Strait Islander (hereafter respectfully referred to as Aboriginal) women in Western Australia participate in BreastScreen WA’s screening mammogram program remains below that for the overall female population of Western Australia. This study aimed to examine perspectives on breast screening amongst Aboriginal women in Western Australia. We explored the factors which impact on participation in breast screening and sought to identify potential initiatives to address lower participation in screening.

**Methods:**

Semi-structured interviews, focus group discussions and yarning sessions were conducted with a total of 65 research participants. They were all Aboriginal and comprised consumers and health professionals from locations across the state.

**Results:**

Our findings show that research participants generally were willing to have a mammogram. Key reasons given were having a genetic predisposition to breast cancer and a perception of investing in health for the sake of the next generation, as well as personal well-being. Barriers identified included lack of education about or understanding of screening, inadequacies in cultural appropriateness in the screening program, cultural beliefs around cancer in general and breast cancer in particular, and competing health and life priorities. However, many enablers were identified which can serve as potential strategies to assuage fear and increase screening uptake. These included increased education delivered by respected Aboriginal women, culturally appropriate promotion and the provision of care and support from other women in the community.

**Conclusion:**

The higher participation rates for Aboriginal women in Western Australia than are found for Aboriginal women nationally demonstrate the success of the strategies put in place by BreastScreen WA. These efforts must be supported and existing policies and practices enhanced to address the limitations in the existing program. Only by implementing and evaluating such initiatives and making breast screening programs more accessible to Aboriginal women can the current disparity between the screening participation rates of Aboriginal and non-Aboriginal women be reduced.

## Background

In keeping with global patterns [1], breast cancer is the most common cancer diagnosed in Australian women [2]. In 2011, there were 7499 new cases of invasive breast cancer diagnosed in Australian women aged 50–69 years. Breast cancer is the second-most common cause of cancer-related death for Australian women with 1126 recorded deaths in 2012 among women aged 50–69 [3]. However, early detection provides an opportunity for treatment which can lead to significantly improved outcomes. BreastScreen Australia aims to reduce morbidity and mortality from breast cancer through early detection using screening mammography [3]. The federally-funded screening program, which operates almost independently of general practice [4], provides free two-yearly screening mammograms to women aged 40 and over, and actively invites women aged 50–74 years to have a screening performed at a screening unit, which may be fixed, relocatable or mobile [3]. Overall participation in BreastScreen Australia’s screening mammogram program has been between 54% and 55% for all years from 2010 to 2011 to 2013–2014 [3].

Breast cancer is also the most common cancer experienced amongst Aboriginal Australian women [5] but there are disparities in incidence and mortality rates between Aboriginal and non-Aboriginal populations. Between 2000 and 2004, in the Australian jurisdictions of New South Wales, Victoria, Queensland (Qld), South Australia (SA) and the Northern Territory (NT) combined, the age-standardised incidence rate of breast cancer was lower for Aboriginal Australian women than for the non-Aboriginal population (84.7 and 115.0 respectively). However, age-standardised mortality rates were higher for Aboriginal women than for non-Aboriginal women. From 2001 to 2005, in Qld, Western Australia (WA), SA and NT combined, the age-standardised mortality rate from breast cancer for Aboriginal women was 26.7 compared with 23.9 for non-Aboriginal Australians [6]. Reasons identified for this disparity in mortality rates include greater likelihood of chronic disease, co-morbidity and barriers to accessing treatment due to geographic remoteness and cultural factors [7–13]. The lower incidence but higher mortality rates of Aboriginal females also suggests that breast cancers are diagnosed at a later stage among Aboriginal females when treatment is not as effective [11, 14]. This later diagnosis is consistent with lower screening rates for Aboriginal Australian women, reported as 36% in 2012–2013 [3].

**Fig. 1:**
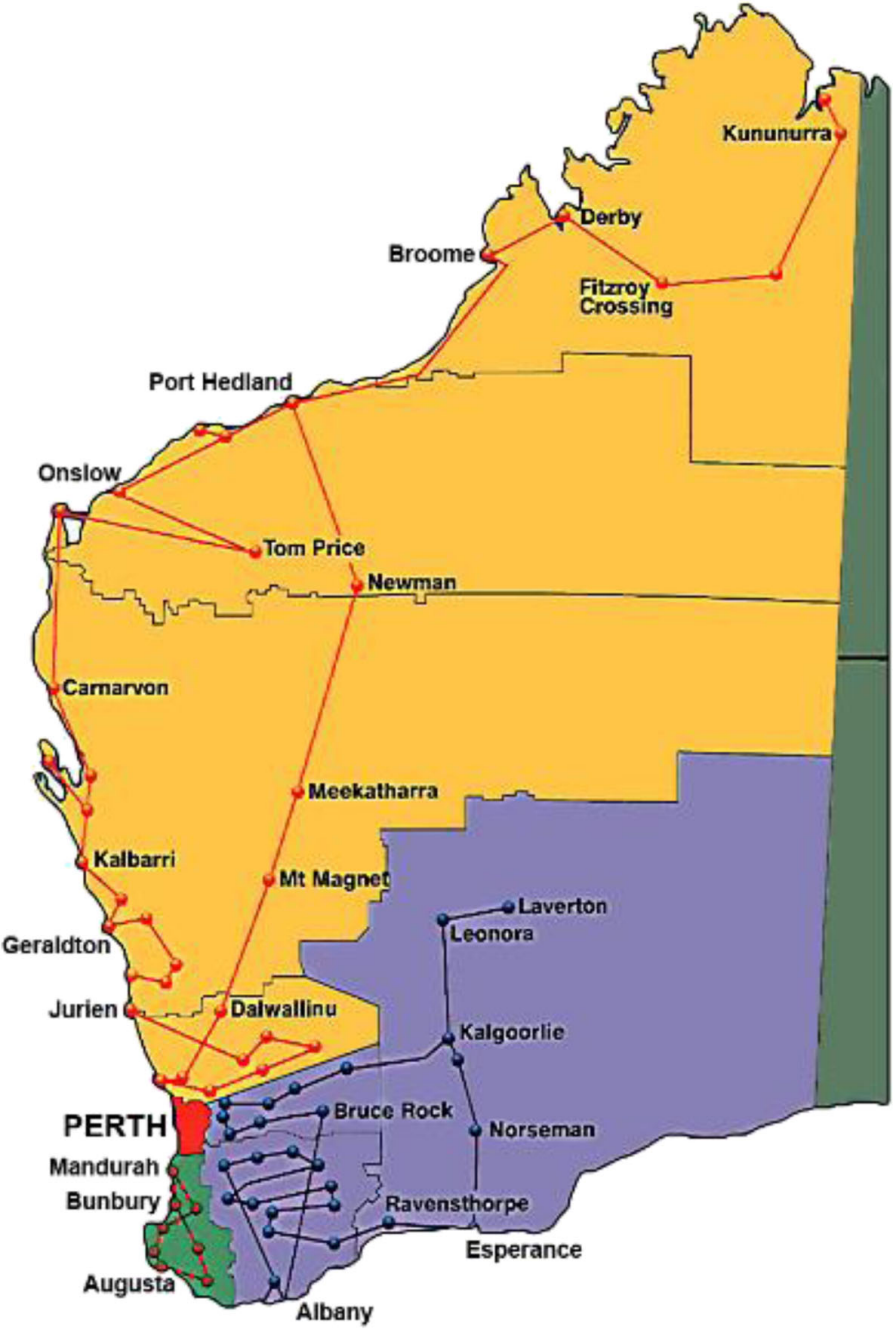
Visiting locations of BreastScreen WA’s mobile service covering the State of Western Australia

WA is Australia’s largest state in terms of land area. In 2015, approximately 79% of WA’s estimated 2.59 million people lived in the state capital, Perth [15]. The state’s population can be differentiated into metropolitan and rural areas, with considerable differences in population density and access to services across the state [16]. It is estimated that 41% of the approximately 88,270 people in WA who identify as Aboriginal live in remote or very remote areas [17]. BreastScreen WA, the agency responsible for breast screening in WA, has been particularly proactive in developing strategies aimed at increasing participation amongst Aboriginal women throughout WA [5]. In 2001, it became one of the first jurisdictional breast screening agencies in Australia to have a dedicated Aboriginal Program Officer. In addition, it provides transport and accommodation assistance to enable women living in remote regions, many of whom are Aboriginal, to access screening facilities. BreastScreen WA has nine screening services located in the Perth-metropolitan area, a regional service based in Bunbury and four mobile vans which visit almost 100 rural towns every two years (Fig. 1 shows the visiting locations of BreastScreen WA’s mobile service) [18]. The service is proactive in promoting its activities and provides training and raises awareness about breast cancer and screening among community groups and organizations which work with Aboriginal women. These strategies may have contributed to higher screening rates for Aboriginal women in WA (44% for 50–69 year olds in 2012–2013) than for Aboriginal women nationally [3]. However, this rate remains below that for the overall female population in WA which stood at 57% in 2012–2013.

The study aimed to examine perspectives on breast screening among Aboriginal women in WA, exploring the factors which impact on their participation in breast screening. It also sought to identify potential initiatives that could consolidate existing efforts to increase participation.

## Methods

### Study design

The study set out to achieve its aims by undertaking qualitative research with information drawn from a cross-section of research participants from metropolitan, rural and remote locations around WA. Groups targeted included Aboriginal women who were eligible for participation in the BreastScreen WA screening program and Aboriginal health professionals. A number of data collection methods were identified – semi-structured interviews, focus group discussions (FGDs) and yarning circles – with the method adopted determined by the needs of research participants. In addition, data collection occurred at locations which were most convenient for research participants.

### Recruitment of research participants

The lead researcher (LP) worked as Aboriginal Program Officer at BreastScreen WA and has an extensive network of Aboriginal health professionals and personal contacts around WA which helped with recruitment of research participants. Details of the research project were emailed to these professional and personal networks who were asked to forward the information to any of their contacts who may be suitable. As in many cases initial contact was not made directly by the lead researcher, the number of potential research participants who were approached and the number who declined to participate could not be determined. However, of the 15 individuals who were approached directly by the lead researcher, 12 agreed to participate in the study.

Two groups of research participants from metropolitan, rural and remote locations around WA were identified for recruitment into the study. The first group consisted of Aboriginal women in the target group for the BreastScreen WA screening program (those aged 40 years and older). These research participants were not required to have previously had a screening mammogram or to have been called to assessment and could include those previously diagnosed with breast or ovarian cancer or who had a family history of breast cancer.

The second group consisted of Aboriginal health professionals involved in women’s health. There were no age or gender restrictions placed on this group. A variety of health professions, including nurses, Aboriginal Health Workers (AHWs), hospital liaison officers and health promotion officers, were targeted. In some instances, research participants belonged to both groups.

### Data collection

Data was collected using semi-structured interviews and FGDs. The FGDs were used to stimulate discussions on breast screening amongst groups of health professionals and consumers. The less formal nature of FGDs removed the constraints that are typical of formal one-on-one interviews and research participants were encouraged to express their views openly knowing there were no right or wrong answers and that different views and experiences to those of others were also encouraged. This mechanism also provided scope for the researcher to probe answers and clarify understandings [19].

“Yarning” was also adopted as a data collection mechanism. This less formal Aboriginal style of oral conversation entails the sharing and exchange of information between two or more people. It can be used in a social context or more formally and can be an appropriate method of information gathering amongst Aboriginal research participants [20]. Yarning was used in situations where the researcher felt that a less structured approach would be more effective and would help to put the research participants at ease.

All research participants were asked about their own experiences of breast screening, if applicable. They were also asked for their understanding of the barriers and facilitators to screening and how these impact on a client’s decision to attend breast cancer screening. Details of the broad domains covered and types of semi-structured interview questions asked are set out in Table 1.

**Table 1:** Semi-Structured Interview Questions

Broad Domain	Questions
Personal experiences of screening	Have you had a screening mammogram? Could you please tell me if you have had a mammogram? If yes, what encouraged you and how did you feel about having one? If not, what prevented you from doing so?
Barriers to screening	What do you think discourages Aboriginal women from having a mammogram? Would you be able to tell me about any cultural or spiritual meanings/ beliefs/ understanding that are attached to cancer among Aboriginal people that might explain why fewer Aboriginal women go for screening? Do you think Aboriginal women know about the breast screening services that are available? What do you think are the existing barriers for Aboriginal women with respect to breast screening services, including access (i.e. distances that have to be travelled, limited times available, etc.), follow-up, cultural, social, financial, any others? Have you heard Aboriginal women discussing the kind of experiences that they have had when dealing with screening services? Please explain by highlighting any experiences that you may have heard of (or may have had personally).
Improvement of services	What do you think could be done to encourage more Aboriginal women to have a mammogram? How do you think the breast screening services should be organized so that they are more culturally sensitive and appropriate and might be better used by Aboriginal women? What else could be put in place to ensure more Aboriginal women access breast screening services?

Informed consent (written or orally recorded) was obtained from all research participants before any interview and/or group discussion was held. For those with literacy difficulties, the information was read aloud and their verbal consent recorded. A small number of research participants provided recorded verbal consent in preference to signing the consent form. In addition, research participants were asked for permission to voice record the interview and/or group discussion to ensure accurate representation of research participant responses. If permission to record was not given, extensive notes were made of the interview.

### Data analysis

Recordings from all data gathering sessions were transcribed verbatim and the transcriptions provided to research participants for checking to confirm their accuracy and, if necessary, to clarify meaning. The interviews which were not voice recorded were “fleshed out” afterwards using interview notes, written up fully and the detailed notes sent to research participants for their approval.

Analysis began with two researchers immersing themselves in the data so they were able to identify and code concepts that emerged from the responses. These were then discussed and reviewed till researchers agreed on key concepts; these were identified as “Research Participants’ Experiences of Breast Screening”, “Barriers to Screening” and “Facilitators to Screening”. For each concept, lower level categories were identified [21]. For example, in the case of barriers to screening, categories such as fear of the results, lack of breast cancer awareness and perceived discomfort associated with the procedure were identified. In working through the analysis, the principle of “describe, compare, relate” was adopted – to describe each category, its characteristics, how research participants spoke about this aspect, and how many mentioned it; to compare differences (if any) in the characteristics for each category between groups; and to relate each category to others to understand the reasons for its occurrence and its consequences [21].

### Cultural governance

To help ensure that the protocols followed were appropriate and respectful of Aboriginal culture and to facilitate the research and knowledge translation, the existing BreastScreen WA Aboriginal Women’s Reference Group, which includes Aboriginal women from all regions of WA, provided cultural advice. In addition, two female elders from the Nyoongar community (Aboriginal Australians whose traditional country is the south-west corner of WA), provided input.

## Results

### Research participants

Many of the consumers contacted through the initial convenience approach were excluded as they did not meet the age criteria for participation. Ultimately, a total of 65 research participants were recruited from whom data was collected using a combination of interviews (12 interviews; 15 research participants); FGDs (two FGDs; 25 research participants) and yarning circles (five yarning circles; 25 research participants). Only those who actively contributed have been included in the totals. A further two individuals who attended a session but participated by indicating their agreement with the opinions of others rather than expressing their own views have not been classified as research participants. There were no differences in profile noted between these individuals and the research participants.

The research participants comprised 59 females and six males and ranged in age from 24 years to 64 years. A profile of the research participants, which outlines further demographic information, is set out in Table 2.

**Table 2:** Research Participant Demographics

Research Participant characteristics	Interviews (n = 12)	Focus Group Discussions (n = 2)	Yarning Circles (n = 5)
Type	3 AHWs (from AMS) 11 Consumers 1 Supporter	25 AHWs (6 from AMS, 19 from CCWA)	6 AHWs (from AMS) 1 AMS Staff member 18 Consumers
Gender	14 Female 1 Male	20 Female 5 Male	All Female
Residence	5 from metropolitan area 5 from regional centres 5 from remote areas	6 AMS AHWs from metropolitan area 19 CCWA AHWs from metro area, four regional centres and six rural/remote centres	7 AMS employees from Regional centre (south of state) 18 Consumers from Remote communities
Location of interview/group discussion	State-wide	CCWA, Perth AMS, Perth	AMS employees - Regional centre (south of state) Consumers - Town in Goldfields region

*AHW* Aboriginal Health Worker, *AMS* Aboriginal Medical Service, *CCWA* Cancer Council WA

#### Interviews

A total of 12 interviews were held at various locations across WA. Fifteen research participants took part in the interviews, three of whom were practicing Aboriginal health professionals. The interviews were all face-to-face, held at a time and place to suit the research participant, often at their home or place of work. The interviewer (LP) was flexible, both in terms of location of interview and with family members attending when this was requested ﻿by the participant.

#### Focus group discussions

A total of two FGDs were held. Both were for Aboriginal health professionals and both took place in Perth.

#### Yarning circles

Five yarning circles were held at regional centres across WA. Three were for Aboriginal health professionals based in a regional centre in the south of the state. A further two were for Aboriginal women who had travelled from remote communities to have a mammogram in a town in the Goldfields-Esperance Region of WA.

### Research participants’ experiences of breast screening

The first concept which emerged from responses was participants’ experiences of breast screening for which findings from the analysis of the constituent categories are presented.

#### Reasons for attending

The majority (53 of the 65) of research participants who took part in the study had experience of having mammograms with two research participants having been diagnosed with breast cancer as a consequence. Many research participants in all groups had their first mammogram before the age of 50 years. Three research participants had gone for a diagnostic mammogram after discovering lumps or experiencing soreness. The other research participants, who had been for screening mammograms, said they went because of a family history of breast cancer, for the sake of their children, for their own benefit, or because it was the “right thing” to do. A number of consumers, who were also health workers, admitted that working in the health profession encouraged them to participate in breast screening, to “practice what you preach”.

#### Influence of others

The influence of others emerged as particularly important in encouraging research participants from remote locations to have a mammogram. Pro-active clinic staff were singled out for their affirmative influence and the term “bossy” was used affectionately a number of times. Other sources of encouragement were family members, friends, and “old timers” (i.e. those who had been for mammograms before). Women from remote locations also said they enjoyed the social element, and the camaraderie experienced during the breast screening outings clearly encouraged these women to participate.

#### Initial fears

Most research participants expressed having no reservations before their first mammogram. Any who were initially apprehensive (mainly women from remote areas) reported that they later realised these fears were unfounded. One research participant (metropolitan location and not a health worker) also felt apprehension before the procedure:

*“I think it is just the fear and also hearing about how painful it is when you are getting your breasts squeezed by that machine.”* (Metropolitan research participant, age 57 years).

#### Ongoing participation

Any trepidation felt initially had generally dissipated. For most research participants, having a mammogram had become routine, facilitated by the accessible and easy BreastScreen WA service, particularly the mobile facility and the social support often provided. Women with a previous cancer diagnosis were found to be more vocal in their support for screening participation than were women who had never been diagnosed with cancer.

### Barriers to screening

The second concept related to barriers to screening.

#### Fear of results

Informants identified fear of the results, combined with fatalistic beliefs around cancer, as the main reason for non-participation by Aboriginal women in screening. This was identified as a barrier by all research participants regardless of their location:

*“You think sometimes it might be because they are scared of finding that they have got breast cancer. I know some women still think that if you get breast cancer it is a death sentence.”* (Remote research participant, age 47 years, AHW).

A number of research participants noted that this fatalistic attitude can prevail in women with a family history of cancer who are often reluctant to have a mammogram because “*they think they’re going to get it [cancer] anyway*.” (Rural research participants, ages 24 and 57 years, AHWs). Seven research participants pointed out that many Aboriginal women would rather not know at all than find out that they have cancer and have to undergo unpleasant treatment when death is seen as inevitable.

#### Lack of knowledge

In addition to feelings of fear around cancer, many research participants described a general lack of knowledge and awareness around breast cancer and screening. One research participant provided a quote which reflected a sentiment she believed is held by many Aboriginal women:

*“There is nothing there, there is no lump there so there is no reason I should go.”* (Remote research participant, age 48 years).

It was felt that many women’s reluctance to have a mammogram is because they are not aware what the process involves. This opinion was most frequently expressed by health workers, both rural and metropolitan, who were aware of challenges with health literacy among some Aboriginal women. Furthermore, it was felt that some women are not aware of the free availability of mammography through the BreastScreen WA program and think that breast screening has to be initiated by a doctor.

#### Discomfort

Although most research participants had no reservations before their first mammogram, they felt that the perceived discomfort associated with the procedure may deter some Aboriginal women from having a mammogram. Fear of the “*squashing down thing*” was identified as a key barrier to participation, particularly by research participants from remote areas.

#### Other priorities

Some research participants expressed the view that many Aboriginal women think that they are just too busy or have other priorities (for example young children) which get in the way of them participating in breast screening. This was predominantly noted by the research participants who were health workers (particularly those based in metropolitan areas), many of whom expressed frustration when women fail to keep appointments and make “excuses” on the day.

#### Shame

“Shame”^1^ was identified as a further barrier to screening. This was raised by all research participants, regardless of age or geographic location. Many Aboriginal women were seen as reluctant to talk about breast cancer and, for some, self-examination is daunting. The idea of participating in breast screening and the ensuing fear of actually having *“their titties out in front of strangers”* (Rural research participant, age 44 years) was highlighted as a significant barrier impeding participation.

#### Other barriers

Other barriers were geographic isolation and lack of transport - these logistical issues were raised more commonly by remote research participants, but recognised even in metropolitan areas. Moreover, even if the service is physically accessible, actually entering “*that place*” causes a problem for some. There can be a distrust of staff in general who are encountered during the screening process, possibly due to a perception that health professionals do not always understand the needs of their Aboriginal clients and how to deal with them appropriately:

*“They don’t really sit there and explain things to you – what the procedure is, why it’s important to do this. They just say ‘Do this’ and that’s it.”* (Metropolitan FGD 1 Research participant, AHW).

### Facilitators to screening

The third concept which emerged from research participants’ responses related to facilitators to screening.

#### Education

Increased education on breast cancer and on the importance of screening and early diagnosis was considered a key to increasing screening participation, a point particularly made by those based in metropolitan and rural but not remote locations. Research participants without a health background emphasised the importance of publicising the work of BreastScreen WA and explaining about the mammography procedure and the wider screening process in ways that are respectful of cultural differences.

Some research participants suggested that information be disseminated in groups, by having a “yarn” although one-on-one sessions should also be available for those not comfortable discussing the topic openly. Education should be carried out ideally by local Aboriginal women who are well regarded in the community, preferably those with personal experience of cancer to increase its saliency**.** Having a local Aboriginal breast cancer survivor, who has endured the tests, treatment and experienced the resulting emotions, was regarded as more powerful for Aboriginal women and more likely to have a positive impact on screening participation:

*“Aboriginal people talking to them … someone that has had breast cancer or something, sitting and talking to people, just sharing, yarning…”.* (Remote research participant, age 48 years).

#### Support

In addition to using local women to provide education, having support from another Aboriginal woman before, during and after the screening process was identified (largely by consumers rather than health professionals) as facilitating participation:

*“…you need someone there really to hold your hand…that support to any Aboriginal woman is crucial from the beginning to the end…” (*Metropolitan research participant),

One easy way to have support readily available was block bookings for Aboriginal women (mentioned a number of times by one AHW research participant) so that women provide moral support to each other during the mammogram process. Alternatively, the education and support could be more formalised, for example, by employing more AHWs to go out into the communities, provide education, encourage women to go for screening and talk them through the process. Whilst the need for education was more likely to be suggested by those in metropolitan and rural locations, those in remote locations identified informal support from other Aboriginal people as particularly important.

#### Mobile van

The mobile van was considered by all groups of research participants to be an effective facilitator of breast screening attendance. Having the van based in an accessible location was recognised as encouraging participation because the requirement to travel may be reduced or eliminated (in remote WA, travel to the van is provided for those living in outlying areas). As one research participant pointed out:

*“If you’ve got the van there, you have got them, there is no excuse.”* (Metropolitan FGD 1 Research participant, AHW).

In addition, the appearance of the van may be considered something of a talking point and generate interest, particularly in a small town:

*“… the clinic is always there, so anyone can go anytime, whereas if the van is there, all the ladies are talking, ‘Oh, the breast screening thing there’ so they sort of encourage each other to go along, you know.”* (Remote research participant, age 48 years).

Timely and effective promotion of the van was considered important to maximise its success. Suggestions from all groups of research participants included promotion in school newsletters aimed at parents and grandparents and in flyers distributed around town and not just at health centres because *“not everybody gets sick”*. Suggested sites for distribution of promotional flyers included shopping centres and Centrelink.

#### Other facilitators

Other suggestions to increase breast screening participation were to widen the transport arrangements to and from the breast screening facility to include metropolitan centres. Health education advertisements on television were identified as a further means of promotion. However, the point was made a number of times (by the health worker research participants) that there are limits to the amount that can be done to encourage participation. Inevitably, each individual is responsible for their own health:

*“We could make it as merry and jolly as you like but can you force someone to have it?”* (Rural research participant, age 56 years, AHW).

## Discussion

This study used qualitative research to examine perspectives on breast screening amongst Aboriginal women in WA. We explored the factors which impact on their participation in breast screening and sought to identify potential initiatives to increase accessibility to Aboriginal women. The results provide a unique insight into the attitudes which Aboriginal women in WA have towards breast screening and, in so doing, it addresses a previously identified gap in Australian research [22].

The research participants indicated a general willingness to have screening mammograms among health workers and consumers alike. A key driver for engagement in breast screening was the perception of investing in health for the sake of the next generation, as well as personal well-being. Recognition of increased risk of breast cancer in the context of a genetic predisposition to the disease also provided impetus for screening participation. Further, health education and group participation appears to have influenced some women on the merits of screening, with some feelings of obligation to participate, possibly as a result of the influence of others. The barriers identified were both structural (for example, lack of education on screening and the absence of cultural appropriateness in the screening program) and social (for example, cultural beliefs around cancer in general, and breast cancer in particular, and competing health and life priorities). Any controversies that may exist in relation to screening and which could influence women’s participation did not emerge as an issue influencing our participants’ willingness to engage in screening. Many enablers were also identified and serve as potential strategies to gradually assuage fear. These included, at a structural level, increased education delivered by respected Aboriginal women, culturally appropriate promotion and greater use of the mobile van service. Social enablers identified were the provision of care and support from other women in the community. While most of the factors identified as barriers or facilitators were mentioned by both groups of participants (health professionals and consumers) this aspect was raised specifically by consumers.

The lower participation of Aboriginal women in mammographic screening is not unique to Australia; participation by Maori women in New Zealand [23, 24], native Hawaiian [25] and American Indian women [26] remains below that of their non-Indigenous counterparts. Furthermore, lower participation by Aboriginal Australians in screening programs is not limited to breast screening. Lower-participation is also reported in the other national population cancer screening programs (the National Cervical Screening Program and the National Bowel Cancer Screening Program) [14]. The challenges associated with engaging Aboriginal women in breast screening programs are complex and manifold. Lower participation cannot be attributed purely to socio-demographic factors [10]. Multiple barriers to participation exist and include individual knowledge and experiences and limitations in the screening program itself [5, 26–28]. To address Aboriginal women’s lower-participation in mammographic screening, it is essential to understand their world views, including their understanding, beliefs and the impediments and facilitators to breast screening.

Lack of understanding about the issues related to breast cancer was a key factor explaining the low participation in breast cancer screening and was a view most commonly expressed by health professionals. This is consistent with low levels of health literacy in relation to cancer generally among Aboriginal people [26, 27]. Linked to this are the misunderstanding and feelings of fear (in particular fatalistic beliefs) and shame which were raised a number of times by research participants. Although, in the context of this research, there was no sense of self-consciousness observed among our research participants who spoke openly about a sensitive subject, some in mixed company, it was acknowledged that amongst the wider Aboriginal population, such feelings may deter some women from participating in screening. These factors can affect decisions around accessing cancer services, including screening [5, 26, 29]. The need to improve health literacy among Aboriginal people in relation to cancer has been recognised elsewhere [26, 27] and was confirmed by our study which identified increased education on breast cancer and screening, appropriate both to cultural differences and literacy levels, as the key facilitator to improve screening participation. Research participants who did not have a background in health care were most commonly of this view. Many materials have been developed and initiatives introduced in locations across Australia [5, 28, 30–33] in efforts to improve breast cancer awareness and deliver early detection messages to Aboriginal women. Some may have resulted in increased numbers of Aboriginal women participating in breast screening programs although more rigorous formal evaluations of efficacy are needed [5, 32]. This reflects a lack of evaluation studies in the Aboriginal health promotion sector in general [34] and the additional challenges typically present in rural/remote and Aboriginal health settings [35, 36].

Although it was not specifically asked, none of the research participants attributed the current deficit in knowledge relating to health matters to lack of available information. Given efforts to improve the content and relevance of information for Aboriginal women, it is likely that this reflects challenges in delivery of this information to women whose lives are filled by other demands. Ineffective dissemination of resources to those responsible for educating and promoting early intervention programs, and a reluctance of these health service providers to fully embrace and carry out their role as educators and promoters of participation in screening programs, may contribute to consumers failing to engage in screening programs [36]. Information on the importance of breast screening must be presented in such a manner as to encourage Aboriginal women to participate. Research participants (in particular, consumers) suggested local women, respected in their community and preferably with personal experience of cancer, as particularly useful to engage women in discussions. Likewise, AHWs were acknowledged as important in providing education, confirming the central role they play in health promotion [37]. BreastScreen WA has achieved considerable success, evidenced by the higher participation rates for Aboriginal women in WA than occur for Aboriginal women nationally. However, its resources are limited and further efforts are needed if access to breast screening programs, across all the dimensions of access recognised by Levesque et al. [38], is to be improved. For this reason, AHWs as key advocates for health promotion must be provided with the necessary support and training to allow them to participate in the effective dissemination of information.

Cancer service providers’ lack of knowledge about the needs of Aboriginal people with cancer has been identified as a major issue impeding communication between patients and health service providers which, in turn, prevents optimal health outcomes [39]. Health professionals, unfamiliar with cultural issues or appropriate responses, can behave in ways that discourage engagement by Aboriginal women in the screening process [5, 26, 28]. Our study found that the approach of staff (including those from Breast Screen WA and from local Aboriginal Medical Services and community clinics), and their use of language may not be appropriate for communicating with Aboriginal people. This point was made both by consumers and health professionals. This may be because staff are unaware of the needs of Aboriginal clients or because they fail to appreciate the anxiety some women feel in a circumstance that they, as health professionals, routinely deal with. It is important that all cancer service providers work respectfully with Aboriginal women and understand the cultural factors that contribute to the acceptance of health messages, particularly in relation to matters around sensitive women’s business^2^ [37]. At a structural level, organisations/health services need to be adequately resourced to provide cultural competence training as part of ongoing professional development to help improve communication and quality of care [39, 40]. A number of cultural awareness training initiatives have been introduced which aim to enhance the understanding of health professionals about the needs of their Aboriginal clients [5, 28, 32, 33, 41]. One study found that Aboriginal clients reported the difference the cultural training had made and felt genuinely welcomed by BreastScreen staff [28]. Others reported success in increasing the numbers of Aboriginal women participating in breast screening programs [32, 41].

The importance of the social context, providing encouragement and support from other women for the entire duration of the screening process, was emphasised as a way of increasing engagement (particularly by consumers) and highlights the notion of peer learning. The provision of informal support has been particularly effective in increasing participation by Aboriginal women in breast screening programs [37]. Influential women, whether clinic staff or from the local community, were considered powerful motivators around participation in screening, particularly for women from remote locations. Elsewhere, lay advisers have been used to navigate the patient through the process [26] and some patient navigator programs have demonstrated increases in adherence to breast screening [42]. Block bookings, which allow women to have their screening done at the same time, are an apparently easy but effective way for Aboriginal women to provide mutual support [28]. Organised transport, with groups of women travelling together to the screening unit was also found to be popular with both groups as useful in encouraging women to participate [28]. Connection to Aboriginal cancer survivors has been found to be of benefit, particularly to those who have been diagnosed with cancer [43]. Research participants in this study suggested that more formal support can be provided by employing AHWs. There is evidence of the supportive role AHWs can play not just for the purpose of breast screening [37] but also in a range of other health care settings [44–46]. The potential for better ways of providing information to Aboriginal women regarding mammography and surviving breast cancer deserves attention.

Providing support is all the more important in the context of the practical barriers Aboriginal women sometimes face. These may impede attendance at screening clinics and include geographical isolation and/or lack of transport, and being busy with other “life priorities” [5]. This highlights the need for flexibility in service delivery, where services adapt to the needs of Aboriginal women to facilitate optimum outcomes in this context. In our study, all groups of research participants recognised the mobile van as a particularly effective way of circumventing these obstacles and providing an accessible service. Providing transport to and from the clinic (particularly for those living in remote regions) has been frequently shown to be another useful facilitator and operates in many locations [5, 28]. Being as flexible and accommodating as possible is vital. One study identified that allowing women to “drop in” or attend as last minute clients was a key facilitator to promoting attendance [28]. The implementation plan recently introduced by the Australian Government, that aims to develop a more flexible health system to better support Aboriginal Australians, demonstrates that the need for adaptability in the delivery of health care is starting to be recognised [47].

It is worth noting that many of the barriers identified relate to structural factors, for example ineffective education on screening and a “one-size-fits-all” model that lacks Aboriginal staff and imposes an inflexible framework [5, 26]. These factors are beyond individual control yet impact on decision making at an individual level. There is clearly a need to review current policies and practices associated with the screening program for their effectiveness in engaging Aboriginal women. A number of recommendations for enhancements to existing policies and practices to make breast screening programs more accessible to Aboriginal women were developed by the authors based on the analysis of the results and are summarised in Table 3. These highlight the key role that partnerships between mainstream services (such as the BreastScreen organisations or the various cancer services) and Aboriginal communities and organisations can play in improving cultural safety, and in so doing increasing accessibility, must not be under-estimated [41, 48, 49]. Such partnerships are integral to involving Aboriginal women in decisions about their health and raising awareness among Aboriginal women of the importance of breast screening and of engaging women in the process.

**Table 3:** Recommendations for increasing accessibility of breast screening programs to Aboriginal women

Logistical • Cater for last minute appointments, or “drop in” sessions without appointment, to increase the flexibility of the service.
• Allocate a block of time reserved for appointments for Aboriginal women to ensure increased cultural security.
• Extend the current transport strategy to include metropolitan and regional centres to create a relaxed and mutually supportive environment, in addition to addressing transport issues.
Cultural • Provide enhanced cultural competence training for BreastScreen staff and clinic staff to improve communication and ensure the provision of optimum care.
• Increase the number of Aboriginal staff at BreastScreen facilities to enhance the acceptability of the service.
• Involve respected, influential Aboriginal women (AHWs or elders) in the screening process to ensure culturally appropriate support before, during and after the screening procedure.
Educational • Increase the number of Aboriginal educators, including local women with an experience of mammography or breast cancer, to provide appropriate information explaining the importance of participation.
• Include Aboriginal breast cancer survivors in support and education to provide a positive message and demonstrate that a cancer diagnosis does not equate to a death sentence.
• Develop resources that can educate and encourage participation of Aboriginal women in mammographic screening, in particular the role women can play in supporting family members and friends to attend.

### Limitations and strengths

Research participants in this study were health professionals and/or those already actively participating in the screening program. Women who are not involved with the program were under-represented. Of those who attended, not all actively contributed to the group discussion and have not been included as research participants. Varying degrees of contribution in group discussions is not unusual, particularly in small Aboriginal community settings where there is limited control over attendance. Furthermore, it is not uncommon for some Aboriginal women not to be very talkative, and a topic such as mammographic screening, which is a part of women’s business, may be more difficult for some women to talk about in group settings. These factors may result in not all those present necessarily being information rich participants. Therefore, the opinions of large segments of the population may not have been captured. But, as representativeness is not the main aim of qualitative research, this is not considered to be a serious limitation. An additional shortcoming may have been the interviewer’s close association with the program which may have prevented some research participants from being as critical of the service as they would otherwise have been.

These recognised limitations must be balanced against the strengths of the study. The research participants for this qualitative research were recruited through multiple means and were drawn from diverse cultural and geographical contexts as shown by the many women from remote communities participating, including some for whom English was not their first language. They provided rich insights into the many reasons for non-participation by Aboriginal women in screening and the triangulation between consumers and health care providers added depth to the study, assisting with validity and study rigour.

The interviewer and lead researcher’s extensive experience of and commitment to working in breast screening programs, in particular her relationship to Aboriginal women, is a strength of this study [50]. The respect with which she is held and her extensive network of Aboriginal health professionals and personal contacts around WA facilitated the recruitment process and subsequent member checking. When interviewed, research participants are likely to have been candid in their responses on the topic because of trust in her, her genuine interest in hearing women’s views and the clear link that existed from findings back into policy and service improvement.

## Conclusion

This study took place in WA where strategies to increase accessibility of breast screening programs to Aboriginal women have been in place for some time. Such are the efforts required that further strategies are necessary to increase access to breast screening programs by Aboriginal women and bridge the gap in attendance rates. Many barriers to participation, such as cultural beliefs around cancer and geographical isolation, are complex and not easily addressed. Processes and approaches that could be adapted to suit the needs of Aboriginal women were identified.

Participation in breast screening programs needs to be made easier and less daunting for Aboriginal women. This cannot be done in isolation but must be carried out as part of an inter-cultural and inter-sectoral partnership to introduce measures at structural and organisational levels to address the limitations in the existing model. Only by implementing such initiatives can the current disparity between the screening participation rates of Aboriginal and non-Aboriginal women be reduced. Increases in screening participation must be matched by culturally appropriate programs that deal with abnormal findings and facilitate Aboriginal women’s pathway through cancer treatment services. Only then will earlier detection of breast cancer translate into better outcomes. Further, the learnings from this study can be applied beyond breast cancer screening to other population-based screening programs, so that the benefits of early detection can be reaped and contribute to closing the gap in health between Aboriginal and non-Aboriginal Australians.

## Footnotes

1 The concept of “shame” as used by Aboriginal English speakers is broader than the non-Aboriginal use of the word. It extends to include embarrassment in certain situations [51] and self-consciousness often due to attention (for positive or negative reasons) rather than as the result of an action by the individual [52].

2 In Aboriginal culture certain customs and practices are performed by men and women separately, often referred to as “Men’s and Women’s Business” [53].
